# Acculturation Strategies and Pap Screening Uptake among Sub-Saharan African Immigrants (SAIs)

**DOI:** 10.3390/ijerph182413204

**Published:** 2021-12-15

**Authors:** Adebola Adegboyega, Jia-Rong Wu, Gia Mudd-Martin

**Affiliations:** College of Nursing, University of Kentucky, Lexington, KY 40536-0232, USA; Jiarong.wu2@uky.edu (J.-R.W.); Gia.mudd@uky.edu (G.M.-M.)

**Keywords:** pap screening, sub-Saharan African immigrants (SAIs), acculturation, cervical cancer screening

## Abstract

Although regular cervical cancer screening can prevent cervical cancer, screening utilization remains low among immigrant population including sub-Saharan African immigrants (SAIs). Acculturation is a complex process, which can lead to adoption of positive or negative health behaviors from the dominant culture. Acculturation strategies are the varying ways in which individuals seek to go about their acculturation by either maintaining or rejecting their own cultural values ip or accepting or rejecting the host culture’s cultural values. Cervical cancer screening behaviors among SAI women may be influenced by their acculturation strategies. We conducted a secondary analysis of data to examine the relationship between acculturation strategies and Pap screening among 99 SAI women recruited from community settings. Data were collected on Pap screening behavior and acculturation strategy. Traditionalists and Integrationists were the dominant acculturation strategies; 32.3% women were Traditionalists and 67.7% Integrationists. From the logistic regression models, Integrationists had seven times the odds of having ever been screened compared to Traditionalists (OR = 7.08, 95% CI = 1.54–28.91). Cervical cancer screening interventions should prioritize Traditionalists for cancer screening. Acculturation strategies may be used to tailor cancer prevention and control for SAIs. More research among a larger SAI women sample is warranted to further our understanding of Pap screening patterns and acculturation strategies.

## 1. Introduction

Cervical cancer is a universal health concern that is treatable if detected early by screening [1]. Racial and ethnic minority women in the United States (U.S.) experience higher risk of cervical cancer compared to other women [2,3,4], which leads to significantly higher incidence and mortality rates [5,6,7,8]. Specifically, the incidence of cervical cancer is 30% higher among Black women compared to their White counterparts and Black women are 80% more likely to die from cervical cancer than White women [9]. Racial and ethnic groups disparities for cervical cancer exist largely because of low socioeconomic status that lead to differences in exposures to risk factors, barriers to high-quality cancer prevention and control, early detection, and treatment [10]. Women who have less educational attainment (high school or less), are uninsured, or do not have a primary care provider have the lowest screening rates [11]. Studies have shown that limited knowledge, fear of screening result, short duration of residence in the U.S., embarrassment, cultural, and religious factors (e.g., fatalism, religiosity etc.) [12,13] are barriers to cervical cancer screening uptake. Barriers associated with the healthcare system and access to care have also been identified including discomfort with the screening procedure [2], cost, inadequate (or lack of) health insurance [8,14], lack of access to screening services, negative past health care experiences, poor patient–provider relationships, and mistrust of the health care system [15]. 

Despite efforts to improve cervical cancer screening rates, the number of eligible Black women who have received Pap screening in the past 3 years remains at 74.8% compared to the Healthy People 2030 target of 84.3% [16,17]. Disparities in cancer care are even greater for immigrants to the U.S. who are less likely to participate in cancer screening than native-born individuals [2,18,19]. For example, Tsui and colleagues found that immigrant women were almost four times as likely as U.S.-born women to have never received a Pap screening (18 vs. 5%) [2]. In addition, a recent literature review of studies conducted in developed countries reported the prevalence of Pap screening ranged from 4.6 to 73.0% among African immigrants [20]. African immigrant women seek cervical cancer screening information from various sources including health care providers, female friends/relatives, and the internet. In the context of cervical cancer screening, verbal presentation of health information is associated with health literacy and information from a health care provider is the most credible source for cervical cancer screening information [21].The U.S. is one of the top migration destinations of sub-Saharan African immigrants living outside of sub-Saharan Africa, with more than 2 million migrants in the U.S. in 2019 [22,23]. In 2015, roughly 39% of sub-Saharan African immigrants (SAIs) had lived in the U.S. for less than ten years, including 19% of whom had arrived within the past four years [23]. As new immigrants to the U.S., SAIs need to adjust and adapt to the environment— including the healthcare system. In fact, migration has been shown to be a critical social determinant of health that contributes to health disparities between immigrants and host communities [24]. Migration can greatly influence women’s preventive health behaviors [23]. Exemplifying this, following resettlement in a host country, women often forgo engagement in health checkups and preventive care to focus on short-term needs such as economic empowerment and language fluency. In addition, studies show that there may be differences in health risk factors and disease profiles between immigrants and host populations [24,25]. For example, SAI women migrate from countries with high prevalence of risk factors for cervical cancer such as high rates of Human Papillomavirus (HPV) infection and low HPV vaccination rates [26,27]. Bruni and colleagues estimated regional-adjusted HPV prevalence at 33.6, 19.6, and 17.4% among Eastern, Western, and Southern sub-Saharan African women, respectively [28]. Despite the high HPV prevalence in sub-Saharan Africa, HPV vaccination coverage rates in this region are among the lowest (1.2% among 10–20-year-old females) globally [29]. In addition, the period of adjustments, adaptations, and acculturation may affect access and use of preventive health care services including cervical cancer screening [24]. Lack of primary care providers, lack of access to screening, and being underinsured or uninsured are common barriers to screening among SAI women [12,13,30].

Acculturation is defined as cultural adaptation and psychological changes that result from a meeting between cultures [31]. The process of acculturation is complex and, in relation to health behaviors, has the potential to lead to adoption of positive or negative behaviors from the dominant culture [32]. For example, although higher acculturation to the U.S. culture has been associated with improved understanding of the importance of cancer screening and increased awareness of preventive screening services, it also could contribute to increased exposure to cancer risk factors such as smoking, physical inactivity, and unhealthy dietary habits [33]. Researchers have reported associations between lower levels of acculturation and decreased use of cancer screening among other immigrant groups in the U.S. [32,34,35,36]. For example, a study among older Chinese Americans found that higher acculturation levels were associated with an increased likelihood of lifetime and current cancer screening completion among Chinese Americans [35]. Similar to other immigrant groups, lower levels of acculturation may be associated with less use of Pap screening among SAIs. Of note, cultural and health beliefs that may be inconsistent with seeking preventive care are common among new and less acculturated immigrants. For instance, the concept of preventive health—such as participating in cervical cancer screening—may be foreign or seen as irrelevant for SAIs, especially if by a woman’s own assessment, she is asymptomatic and appears otherwise healthy [37].

Acculturation strategies refer to varying ways in which individuals approach acculturation [38,39].The development of acculturation strategies are based on two important issues individuals must confront in their daily life. The first issue is whether to maintain or reject their own cultural values, while the second is whether to accept or reject the host culture’s cultural values [31]. According to Berry [31], acculturation strategies depend on the extent to which an individual balances the issues of their heritage culture maintenance and wishes to have contact with those who are outside their group and participate with them in the daily life of the larger society. For this study, we used Berry’s classification to classify women’s acculturation strategies into four groups [38,39]. Berry identified four acculturation strategies: (a) Traditionalist, (b) Integrationist, (c) Assimilationist, and (d) Marginalist. A Traditionalist resists acculturation, chooses not to identify with another culture, and retains separate ethnic identification, behaviors, beliefs, practices, and values. An Integrationist develops a bicultural orientation by simultaneous integration and identification with both cultures and being comfortable with both groups [31]. An Assimilationist rejects their own cultural values and identity and accept the host cultural values while a Marginalist distances themself from the original culture for identification with another culture only to be rejected by the new culture [31]. The Marginalist therefore no longer identifies with either culture [31]. 

Previous evidence for the association between acculturation and cancer screening has been reported [32,34,35,40]. Acculturation to U.S. customs over time has been shown in some studies to facilitate engagement in cancer screening on a regular basis [41]. In a study among Hispanic women [40] that used language as a measure of acculturation, lower acculturated women (women with lowest summed score on language questions) were less likely to have ever had a Pap screening, or to have been screened in either the past 3 years or the past year. Additionally, the odds of having ever been screened increased with increasing level of acculturation [40]. In a study among Chinese Americans, Li and colleagues who measured acculturation with the Population Study of Chinese Elderly (PINE) study acculturation scale, observed that higher acculturation levels were associated with an increased likelihood of lifetime and current cancer screening among older Chinese Americans [35]. 

There is limited research regarding the association between acculturation and Pap screening uptake among SAI women in the U.S. Understanding the role that acculturation plays in Pap screening among SAI women is important in order to promote screening and fully realize the benefits of Pap screening for the reduction in cervical cancer morbidity and mortality. We examined the relationship between acculturation strategies and Pap screening among SAIs. We hypothesized that women who were Integrationists (women with bicultural orientation) were more likely to have had a Pap screening compared with Traditionalists. An understanding of the association between acculturation and Pap screening can inform tailored interventions to promote cervical health for SAIs and other immigrant women in the U.S.

## 2. Materials and Methods

### 2.1. Study Design

This is a secondary analysis of data from a cross-sectional study conducted to examine correlates of Pap screening among 108 SAI women [42]. Participants who were female, age 21 or above, self-identified as sub-Saharan African immigrants, had no history of hysterectomy, could read and write in English, and resided in Kentucky were eligible to enroll in the study. Women were recruited from urban locations in the state using purposive and snowballing sampling methods between October 2016 and January 2017. Participants completed a self-administered questionnaire on paper or a password-protected iPad designated for the study. Among participants in the original study, 65.7% reported ever having had Pap screening, 29.6% reported never having been screened, and 4.6% reported that they did not know whether they had ever been screened. Results indicated that provider’s recommendation and Pap screening awareness were significantly associated with receiving Pap screening [42]. Research procedures and outcomes have been previously published [42].

For the purposes of this study, 99 participants for whom complete data on acculturation, Pap screening, and controlled variables (age, health insurance, income, education, and provider’s recommendation for Pap) were available were included in the analyses. The majority of participants were either Traditionalists or Integrationists; therefore, this analysis was limited to participants in either of these strategies. The University of Kentucky Institutional Review Board approved all research procedures prior to research commencement.

All participants completed a standard sociodemographic questionnaire used to gather data on age, country of origin, income, financial comfort, provider recommendation for pap screening, insurance status, education, marital status, and employment status. 

*Financial comfort*: Financial comfort was assessed with the question, “Considering the amount of money that comes into your household for you to live on, would you say that you are: (a) very financially comfortable, having more than enough to make ends meet; (b) financially comfortable, having enough to make ends meet; or (c) not financially comfortable because you do not have enough to make ends meet. The values were recoded into a dichotomous variable with responses categorized as “not financially comfortable” or “financially comfortable”, with the latter representing those who self-identified as “very financially comfortable” or “financially comfortable”. Although data on income were reported, financial comfort has strong association with engagement in health protective behaviors [43,44] and was therefore used in our analysis. 

*Outcome variable--Pap screening*: To assess ever having had a Pap screening, participants were asked if they had ever had a Pap screening, with response options of “yes”, “no”, or “don’t know”. Women who answered, “don’t know” were classified with women who answered no to dichotomize the response.

*Independent variable--Acculturation strategy*: Participant’s acculturation strategies were calculated as composite mean score from the modified psychological acculturation scale [45]. Behavioral acculturation scale [46], and Cultural identity (described below) [46]. The acculturation instrument assessed beliefs and behaviors along two dimensions; D1: relative preference for maintaining the African ethnocultural group and D2: relative preference for having contact with and participating in American culture [46]. The scores obtained from D1 and D2 were then used to classify participants into four acculturation strategies: (a) Traditionalist, (b) Integrationist, (c) Assimilationist, and (d) Marginalist. The Cronbach’s alphas for D1 and D2 were 0.94 and 0.88, respectively, in a study among Nigerian and Ghanaian born-immigrants in the Baltimore–Washington D.C. area [46]. In this study, the Cronbach’s alphas for D1 and D2 were 0.90 and 0.88, respectively.

*The modified psychological acculturation scale* was used to measure psychological aspect of acculturation. The original Psychological Acculturation Scale was developed by Tropp et al. [45] to assess an individual’s sense of emotional attachment to, belonging within, and understanding of the Anglo American and Latino–Hispanic cultures. The original instrument included 10 items focused on individual’s psychological responses to differing cultural contexts. In the modified version, items were applied to both the U.S. and African culture and were rated on a 5-point Likert scale ranging from “strongly disagree to strongly agree” [46]. For example, “I share most of my beliefs and values with African people” and “I share most of my beliefs and values with American people”.

*Behavioral acculturation scale:* Behavioral acculturation was measured using the 2-item Behavioral acculturation scale. Participants were asked how often they spent time with American/African people and items were rated from “never” to “always”. Participants were also asked how many American/African friends they had and their responses ranged from “none” to “very many” [46,47].

*Cultural identity*: We assessed cultural identity with the 2 items “I feel American” and I feel African”. Items were rated on a 5-point Likert scale ranging from “strongly disagree” to “strongly agree” [46,47].

### 2.2. Data Analyses

Descriptive statistics, including means and SD or frequency distributions, were used to characterize participants. Logistic regression analysis was conducted to assess if acculturation strategies were associated with Pap screening. Variables most often associated with Pap screening in the general U.S. population [11,26] as well as immigrant populations [20,41,48] were controlled for in the logistic regression model, including age, education, financial comfort, length of residence in the U.S., health insurance, and provider’s recommendation. All analyses were conducted using IBM SPSS Statistics for Windows, Version 26.0. ((IBM Corp, Armonk, NY, USA) with an alpha of *p* < 0.05 set a priori as indicative of statistical significance. 

## 3. Results

### 3.1. Participant Characteristics

The sample included responses from 99 women. Descriptive characteristics are presented in Table 1. Participants had mean age 34.3 (SD—9.1) years. Sixty-seven percent reported ever having had a Pap screening. Sixty three percent were insured, majority had college education and were financially comfortable, and about one quarter had household income above USD 35,000 and 20 women selected “do not know” as the response for income. The 99 SAI women included in this analysis represented 14 different countries of origin. The top five countries represented were Nigeria (35.4%), Cameroon (23.2%), Ghana (12.1%), Congo (10.1%), and Kenya (6.1%). 

### 3.2. Bivariate Analyses

Regarding acculturation strategy, 32.3% of the women were traditionalist and 67.7% integrationist. Traditionalist and integrationist groups were compared using independent samples t-tests for continuous variables and chi square analyses for categorical variables (Table 1). Integrationists were younger (mean age—32.87, S.D—8.4) than Traditionalists (mean age—37.44, S.D—9.7), *p* = 0.018, and reported higher financial comfort than Traditionalists (73.1 vs. 49.5%, *p* = 0.020). There were no significant differences in educational level, marital status, length of U.S residence, and health insurance status. 

***Logistic regression analyses***. In the adjusted analysis with acculturation strategy as the main independent variable, compared to Traditionalists, Integrationists were seven times (95% CI = 1.74–28.91, *p* = 0.006) more likely to have ever had a Pap screening (see Table 2). We also found two covariates, financial comfort and provider’s recommendation for Pap screening, were associated with a Pap screening. Women who reported financial comfort had lower odds (95% CI: 0.04–0.525, *p* = 0.003) of reporting a Pap screening compared with those who reported financial discomfort. Women who had received a recommendation from their provider to have a Pap screening compared to those who had not received a recommendation had greater odds (95% CI: 5.12–157.73) of having had Pap screening. While these were statistically significant, these odds ratio should be cautiously interpreted given the sample size and large confidence intervals.

## 4. Discussion

The primary objective of this study was to examine the relationship between acculturation strategies and Pap screening among SAI women. We observed two major acculturation strategies among SAI women in this study: Integrationists and Traditionalists. Women in this study who were Integrationists were more likely to report ever having had a Pap screening than Traditionalists. Integrationists blend both cultures’ attitudes, beliefs, and/or identities [49]. LaFromboise and colleagues have speculated that individuals who are bicultural develop skills related to identity, language, knowledge of cultural beliefs and values, as well as effective support systems in both cultures that supports better physical and mental health [50]. As women who are Integrationists spend more time in the U.S., it is likely therefore that they become increasingly aware of cancer screening and prevention services available to them through the healthcare system [41] and become more enlightened about how to better navigate the complex system. Other factors that could explain women’s increased probability to get screened include having access to a primary care provider, patient–provider gender concordance, increased length of residence in the U.S., immigration status, affordability, Pap screening knowledge, health provider recommendation regarding Pap screening, and health literacy [20,30,42]. Notably, Integrationists in our study were younger and more financially comfortable than Traditionalists, two factors that could also impact likelihood of receiving Pap screening. 

These findings are important for our understanding of associations between acculturation and cancer screening practices, as there are relatively few studies in which associations between acculturation strategies and cancer screening are examined. In fact, much of the existing literature uses proxy measures of acculturation (language, nativity status, length of residence, etc.). While still others use validated measures of acculturation, most categorize individuals as either acculturated or not, or as having low, moderate, and high acculturation. [32,34]. Rather than examining acculturation as a categorical variable, the findings of this study serve to advance our understanding of the complexities of acculturation by examining associations between acculturation strategies and Pap screening uptake among SAI.

Our outcomes support the findings from other studies that have shown that a provider’s recommendation to have Pap screening is significantly associated with cancer screening. This finding highlights the importance of having access to a health care provider who can recommend cancer screening and appropriate follow-up. Beyond access, providers’ willingness to discuss screening guidelines, benefits and risks of screening, and screening options for cervical cancer can greatly impact women’s engagement in Pap screening [42]. 

Our finding that individuals that reported financial comfort had decreased odds of having ever had a Pap screening was not in accord with previous studies [41,51]. In a qualitative study, Ndukwe and colleagues reported that costs and lack of insurance are barriers to Pap screening among SAI women [51]. Even though high socioeconomic status is associated with cancer screening [8,52], financial comfort alone may not guarantee health care access to cancer screening. Other determinants of access may have a more significant impact on screening practices among SAI women. According to Penchansky and Thomas’ conceptualization of access, health care access reflects the “fit” between health care consumers and the health care system [53]. Penchansky and Thomas [53] group access into five A’s: affordability—health care costs; availability—resources to meet patients’ needs; accessibility—ease of physical access to the health care system; accommodation—the extent to which the health care system meets the patient’s needs and preferences, and acceptability—the relationship of the patient’s and health care provider’s attitudes, preferences, and characteristics related to each other. The dimensions of access are not easily separated. For example, it is plausible that individuals reporting financial comfort are in the working population who may not have time to get screened or may not be employed in positions in which health insurance is offered. The lack of clinic schedules compatible with the work schedules of women in the service sector have been reported as a barrier to screening among immigrant women [2]. The finding from our study requires further research in order to have a comprehensive understanding of other determinants of health care access and multiple influences on screening uptake among SAI women 

Further exploration of Pap screening patterns and acculturation strategies among a larger sample of SAI women is warranted. This would support greater understanding of associations between acculturation and engagement in cervical cancer screening behaviors. Considering the growing size of SAIs in the U.S, tailored health interventions can provide a foundation for developing intervention approaches appropriate for the varying acculturation strategies for SAI women that can lead to early detection and prevention of cervical cancer.

### Study Limitations

Although our findings provided important data regarding associations between acculturation strategies and Pap screening, results should be interpreted with consideration of study limitations. Temporal relationships cannot be inferred and results cannot be generalized to other SAI women because of the use of convenience sampling and cross-sectional design. Another limitation was the small sample size resulting in wide confidence intervals for the adjusted results due to increased estimate variability. In addition, women self-reported prior completion of Pap screening, which may be subject to recall or social desirability bias. It is also important to recognize that SAI populations are heterogeneous, including people from many countries and cultures. The majority of women in this study reported college and post-graduate education as their highest education. At the same time, characteristics—such as educational level of our sample—reflect national data from the Pew Research Center that suggests sub-Saharan immigrants in the U.S. are more highly educated than the overall U.S.-born population. [22]. In order to optimize interventions aimed at improving Pap screening behaviors among SAI women, larger studies using robust sampling strategies to ensure representative subgroups of SAI women are needed.

## 5. Conclusions

Our study contributes to the literature by examining associations between acculturation strategies and Pap screening among SAI women. Understanding the relationship between acculturation strategies adopted by SAI women and Pap screening uptake may provide insight into the development of behavioral interventions to increase Pap screening for SAI women. Additionally, enhanced understanding of the complexities of acculturation by considering varying strategies can contribute to new perspectives on approaches to cancer prevention not only for SAI women but for other immigrant groups. This could lead to more effective prevention and control interventions with the potential to reduce disparities associated with cervical cancer screening and rates of cervical cancer among immigrant groups.

## Figures and Tables

**Table 1:** Sample characteristics by acculturation strategy (N = 99).

Variable	Total N = 99 Mean ± SD; N (%)	Traditionalists N = 32 Mean ± SD; N (%)	Integrationists N = 67 Mean ± SD; N (%)	*p* Value
Age in years	34.3 ± 9.1	37.4 ± 9.7	32.9 ± 8.4	0.018
Education in years	14.3 ± 6.6	12.3 ± 6.8	15.0 ± 6.4	0.129
Marital status				0.189
Not married	34 (34.3) 53 (53.5) 12 (12.1)	7 (21.9) 20 (62.5) 5 (32)	27 (40.3) 33 (49.3) 7 (10.4)	
Married/cohabitating				
Separated/divorced/widowed				
Length of U.S. residence				0.321
≤5 years >5 years	47 (47.5) 52 (52.5)	18 (56.3) 14 (43.8)	29 (43.3) 38 (56.7)	
Income				0.309
≤35,000 >35,000 Don’t know	54 (54.5) 25 (25.3) 20 (20.2)	20 (37.0) 5 (20.0) 7 (35.0)	34 (63.0) 20 (80.0) 13 (65%)	
Health insurance No health insurance	63 (63.1) 36 (36.4)	19 (59.4) 13 (40.6)	44 (65.7) 23 (34.3)	0.700
Financial comfort Financial discomfort	64 (64.6) 35 (35.4)	15 (49.5) 17 (53.1)	49 (73.1) 18 (26.9)	0.020

**Table 2:** Logistic regression model for the association between acculturation strategy and Pap Screening (N = 99).

Characteristics.	B	S.E	Odds Ratio (95% CI)	*p* Value
***Independent variable***				
**Acculturation strategy**				0.006
Traditionalists (Ref)			1.0	
Integrationists	1.957	0.718	7.08 (1.74–28.91)	
***Covariates***				
Age (years)	0.027	0.033	1.03 (0.96–1.10)	0.404
Education (years in school)	−0.002	0.041	0.99 (0.92–1.08)	0.964
**Length of stay in the U.S.**				0.096
≤5 years (Ref)			1.0	
>5 years	1.123	0.675	3.07 (0.82–11.54)	
**Insurance Status**				0.170
Not insured (Ref)			1.0	
Insured	0.915	0.667	2.50 (0.68–9.22)	
**Financial comfortability**				0.003
Financial discomfort (Ref)			1.0	
Financial comfort	−1.982	0.667	0.14 (0.04–0.52)	
**Provider recommendation for Pap**				<0.001
No provider recommendation (Ref)			1.0	
Provider recommendation	3.347	0.874	28.42 (5.12–157.73)	

## Data Availability

The data that support the findings of this study are available from the corresponding author, upon reasonable request.

## References

[1] National Center for Chronic Disease Prevention and Health Promotion CDC Vital Signs. *Cervical Cancer Is Preventable*. https://www.cdc.gov/vitalsigns/cervical-cancer/index.html.

[2] Tsui J., Saraiya M., Thompson T., Dey A., Richardson L. (2007). Cervical Cancer Screening among Foreign-Born Women by Birthplace and Duration in the United States. J. Womens Health.

[3] Lin L., Benard V.B., Greek A., Hawkins N.A., Roland K.B., Saraiya M. (2015). Racial and ethnic differences in human papillomavirus positivity and risk factors among low-income women in Federally Qualified Health Centers in the United States. Prev. Med..

[4] Musselwhite L.W., Oliveira C.M., Kwaramba T., Pantano N.D.P., Smith J.S., Fregnani J.H., Reis R.M., Mauad E., Vazquez F.D.L., Longatto-Filho A. (2016). Racial/Ethnic Disparities in Cervical Cancer Screening and Outcomes. Acta Cytol..

[5] Rositch A.F., Nowak R.G., Gravitt P.E. (2014). Increased age and race-specific incidence of cervical cancer after correction for hysterectomy prevalence in the United States from 2000 to 2009. Cancer.

[6] Patel D.A., Barnholtz-Sloan J.S., Patel M.K., Malone J.M., Chuba P.J., Schwartz K. (2005). A population-based study of racial and ethnic differences in survival among women with invasive cervical cancer: Analysis of Surveillance, Epidemiology, and End Results data. Gynecol. Oncol..

[7] Beavis A.L., Gravitt P.E., Rositch A. (2017). Hysterectomy-corrected cervical cancer mortality rates reveal a larger racial disparity in the United States. Cancer.

[8] DeSantis C.E., Siegel R.L., Sauer A.G., Miller K.D., Fedewa S.A., Alcaraz K.I., Jemal A. (2016). Cancer statistics for African Americans, 2016: Progress and opportunities in reducing racial disparities. CA A Cancer J. Clin..

[9] American Cancer Society Cancer Facts & Figures for African Americans 2019–2021.

[10] Ward E., Jemal A., Cokkinides V., Singh G.K., Cardinez C., Ghafoor A., Thun M. (2004). Cancer Disparities by Race/Ethnicity and Socioeconomic Status. CA A Cancer J. Clin..

[11] Sauer A.G., Bandi P., Saslow D., Islami F., Jemal A., Fedewa S.A. (2020). Geographic and sociodemographic differences in cervical cancer screening modalities. Prev. Med..

[12] Ekechi C., Olaitan A., Ellis R., Koris J., Amajuoyi A., Marlow L.A. (2014). Knowledge of cervical cancer and attendance at cervical cancer screening: A survey of Black women in London. BMC Public Health.

[13] Adegboyega A., Hatcher J. (2016). Factors Influencing Pap Screening Use among African Immigrant Women. J. Transcult. Nurs..

[14] Nolan J., Renderos T.B., Hynson J., Dai X., Chow W., Christie A., Mangione T.W. (2014). Barriers to Cervical Cancer Screening and Follow-up Care among Black Women in Massachusetts. J. Obstet. Gynecol. Neonatal Nurs..

[15] Weragoda J., Azuero A., Badiga S., Bell W.C., Matthews R., Piyathilake C. (2016). An examination of racial differences in 5-year survival of cervical cancer among African American and white American women in the southeastern US from 1985 to 2010. Cancer Med..

[16] Healthy People 2030. https://health.gov/healthypeople/objectives-and-data/browse-objectives/cancer/increase-proportion-females-who-get-screened-cervical-cancer-c-09.

[17] National Cancer Institute Cervical Cancer Screening. Cancer Trends Progress. https://progressreport.cancer.gov/detection/cervical_cancer.

[18] Cofie L.E., Hirth J.M., Wong R. (2018). Chronic comorbidities and cervical cancer screening and adherence among US-born and foreign-born women. Cancer Causes Control..

[19] Consedine N.S., Tuck N.L., Ragin C.R., Spencer B.A. (2014). Beyond the Black Box: A Systematic Review of Breast, Prostate, Colorectal, and Cervical Screening among Native and Immigrant African-Descent Caribbean Populations. J. Immigr. Minor. Health.

[20] Cudjoe J., Nkimbeng M., Turkson-Ocran R.-A., Commodore-Mensah Y., Han H.-R. (2020). Understanding the Pap Testing Behaviors of African Immigrant Women in Developed Countries: A Systematic Review. J. Immigr. Minor. Health.

[21] Cudjoe J., Gallo J.J., Sharps P., Budhathoki C., Roter D., Han H.-R. (2021). The Role of Sources and Types of Health Information in Shaping Health Literacy in Cervical Cancer Screening Among African Immigrant Women: A Mixed-Methods Study. HLRP Health Lit. Res. Pr..

[22] Echeverria-Estrada C., Batalova J. (2019). Sub-Saharan African Immigrants in the United States. https://www.migrationpolicy.org/article/sub-saharan-african-immigrants-united-states-2018.

[23] Anderson M., Connor P. (2018). Sub-Saharan African Immigrants in the U.S. Are often More Educated than Those in Top European Destinations.

[24] Castañeda H., Holmes S.M., Madrigal D.S., Young M.E., Beyeler N., Quesada J. (2015). Immigration as a social determinant of health. Annu. Rev. Public Health.

[25] Giuntella O., Kone Z., Ruiz I., Vargas-Silva C. (2018). Reason for immigration and immigrants’ health. Public Health.

[26] Forney-Gorman A., Kozhimannil K. (2015). Differences in Cervical Cancer Screening Between African-American versus African-Born Black Women in the United States. J. Immigr. Minority Health.

[27] Vaccarella S., Laversanne M., Ferlay J., Bray F. (2017). Cervical cancer in a frica, Latin a merica and the C aribbean and a sia: Regional inequalities and changing trends. Int. J. Cancer.

[28] Bruni L., Diaz M., Castellsagué X., Ferrer E., Bosch F.X., José F.X.B. (2010). Cervical Human Papillomavirus Prevalence in 5 Continents: Meta-Analysis of 1 Million Women with Normal Cytological Findings. J. Infect. Dis..

[29] Bruni L., Diaz M., Barrionuevo-Rosas L., Herrero R., Bray F., Bosch F.X., de Sanjosé S., Castellsagué X. (2016). Global estimates of human papillomavirus vaccination coverage by region and income level: A pooled analysis. Lancet Glob. Health.

[30] Adegboyega A., Aleshire M., Linares A.M. (2017). Examining Cervical Cancer Screening Utilization among African Immigrant Women: A Literature Review. Int. J. Womens Health Wellness.

[31] Sam D.L., Berry J.W. (2010). Acculturation: When individuals and groups of different cultural backgrounds meet. Perspect. Psychol. Sci..

[32] Nguyen A.B., Clark T.T. (2014). The Role of Acculturation and Collectivism in Cancer Screening for Vietnamese American Women. Heal. Care Women Int..

[33] Medhanie G.A., Fedewa S.A., Adissu H., DeSantis C.E., Siegel R.L., Jemal A. (2017). Cancer incidence profile in sub-Saharan African-born blacks in the United States: Similarities and differences with US-born non-Hispanic blacks. Cancer.

[34] Tejeda S., Gallardo R.I., Ferrans C.E., Rauscher G.H. (2016). Breast cancer delay in Latinas: The role of cultural beliefs and acculturation. J. Behav. Med..

[35] Li C.-C., Matthews A.K., Dong X. (2018). The Influence of Health Literacy and Acculturation on Cancer Screening Behaviors among Older Chinese Americans. Gerontol. Geriatr. Med..

[36] Johnson-Kozlow M. (2010). Colorectal cancer screening of Californian adults of Mexican origin as a function of acculturation. J. Immigr. Minority Health.

[37] Ogunsiji O.O., Kwok C., Fan L.C. (2017). Breast cancer screening practices of African migrant women in Australia: A descriptive cross-sectional study. BMC Womens Health.

[38] Berry J.W. (1992). Acculturation and Adaptation in a New Society. Int. Migr..

[39] Berry J.W. (2005). Acculturation: Living successfully in two cultures. Int. J. Intercult. Relat..

[40] Shah M., Zhu K., Wu H., Potter J. (2006). Hispanic acculturation and utilization of cervical cancer screening in the US. Prev. Med..

[41] Adunlin G., Cyrus J., Asare M., Sabik L. (2018). Barriers and Facilitators to Breast and Cervical Cancer Screening Among Immigrants in the United States. J. Immigr. Minor. Health.

[42] Adegboyega A., Dignan M., Hatcher J. (2019). Correlates of pap screening among sub-saharan immigrant women. J. Health Care Poor Underserved.

[43] Mudd-Martin G., Rayens M.K., Lennie T.A., Chung M.L., Ms Y.G., Wiggins A.T., Biddle M.J., Bailey A.L., Novak M.J., Casey B.R. (2014). Fatalism Moderates the Relationship Between Family History of Cardiovascular Disease and Engagement in Health-Promoting Behaviors Among At-Risk Rural Kentuckians. J. Rural. Health.

[44] Harley A.E., Yang M., Stoddard A.M., Adamkiewicz G., Walker R., Tucker-Seeley R.D., Allen J.D., Sorensen G. (2014). Patterns and Predictors of Health Behaviors among Racially/Ethnically Diverse Residents of Low-Income Housing Developments. Am. J. Health Promot..

[45] Tropp L.R., Erkut S., Coll C.G., Alarcón O., García H.A.V. (1999). Psychological Acculturation: Development of A New Measure for Puerto Ricans on the U.S. Mainland. Educ. Psychol. Meas..

[46] Commodore-Mensah Y., Ukonu N., Cooper L.A., Agyemang C., Himmelfarb C.D. (2018). The association between acculturation and cardiovascular disease risk in Ghanaian and Nigerian-born African immigrants in the United States: The Afro-Cardiac Study. J. Immigr. Minority Health.

[47] Commodore-Mensah Y., Sampah M., Berko C., Cudjoe J., Abu-Bonsrah N., Obisesan O., Agyemang C., Adeyemo A., Himmelfarb C.D. (2016). The afro-cardiac study: Cardiovascular disease risk and acculturation in west African immi-grants in the United States: Rationale and study design. J. Immigr. Minority Health.

[48] Maxwell A.E., Bastani R., Warda U.S. (2000). Demographic predictors of cancer screening among Filipino and Korean immigrants in the United States. Am. J. Prev. Med..

[49] Chen S.X., Benet-Martínez V., Bond H.M. (2008). Bicultural Identity, bilingualism, and psychological adjustment in multicultural societies: Immigration-based and globalization-based acculturation. J. Personal..

[50] LaFromboise T., Coleman H.L., Gerton J. (1993). Psychological impact of biculturalism: Evidence and theory. Psychol. Bull..

[51] Ndukwe E.G., Williams K.P., Sheppard V. (2013). Knowledge and perspectives of breast and cervical cancer screening among female African immigrants in the Washington D.C. metropolitan area. J. Cancer Educ..

[52] American Cancer Society Cervical Cancer Prevention and Screening: Financial Issues.

[53] Penchansky R., Thomas J.W. (1981). The concept of access: Definition and relationship to consumer satisfaction. Med. Care.

